# High Risk of Revision Associated with the L-Cup Titanium Alloy Porous Coated Acetabular Component in Primary Total Hip Arthroplasty: Minimum Follow-Up of 14 Years

**DOI:** 10.3390/jcm14041301

**Published:** 2025-02-15

**Authors:** Marek Drobniewski, Kacper Ruzik, Bartosz Gonera, Łukasz Olewnik, Adam Borowski, George Triantafyllou, Andrzej Borowski

**Affiliations:** 1 Clinic of Orthopaedic and Paediatric Orthopaedics, Medical University of Lodz, 90-419 Łódź, Poland; marek.drobniewski@umed.lodz.pl (M.D.); andrzej.borowski@umed.lodz.pl (A.B.); 2 Department of Clinical Anatomy, Masovian Academy in Płock, 09-402 Płock, Polandlukaszolewnik@gmail.com (Ł.O.); 3 Medical University of Warsaw, 02-091 Warsaw, Poland; adamborowskio15@gmail.com; 4 Department of Anatomy, School of Medicine, National and Kapodistrian University of Athens, Goudi, 12462 Athens, Greece; georgerose406@gmail.com

**Keywords:** primary total hip arthroplasty, coxarthrosis, orthopedic surgery, acetabular component

## Abstract

**Background:** Hip joint pain due to arthritis is a prevalent issue in adults, often necessitating surgical intervention such as total hip arthroplasty (THA). This procedure has been celebrated for its reliability; however, successful outcomes depend on numerous factors. Current advancements are focused on improving implant design and surgical methodologies. This study aimed to evaluate the long-term clinical and functional outcomes of uncemented total hip arthroplasty utilizing the L-Cup acetabular component. **Methods:** Between February 1999 and November 2010, 351 L-Cup components were implanted in 315 patients. A follow-up period ranged from 14 to 25 years. The clinical outcomes were assessed using the modified Merle d’Aubigné and Postel (MAP) classification and patient satisfaction was measured using a Visual Analog Scale (VAS). **Results:** Postoperative evaluations showed significant improvement, with VAS scores decreasing from a mean of 7.2 to 2.1, indicating substantial pain alleviation. The modified MAP classification showed a significant improvement of 6.3 points throughout the follow-up period. The results revealed that 49.5% of the cases were classified as excellent, while 20.5% had poor outcomes due to prosthesis loosening. According to the Kaplan–Meier estimator, the 5-year survival rate for the acetabular component was 97.78%, with survival rates of 90.5% at 10 years, 80.45% at 15 years, and 73.79% at 20 years. **Conclusions:** Total hip arthroplasty is an effective treatment for advanced degenerative joint diseases. While significant postoperative improvements were documented, the observed prosthesis loosening in 20.5% of cases raises concerns about the long-term effectiveness of the L-Cup acetabular component and suggests the need for further refinement in surgical techniques and implant design.

## 1. Introduction

Hip joint pain resulting from arthritis is one of the most prevalent reasons for medical consultations in the adult population. Due to the progressive course of the disease, it requires a specific approach and appropriate classification for the type of treatment [1]. Severe osteoarthritis of the hip joint is characterized by increasing pain symptoms and limited mobility. In cases where pharmacological conservative treatment and rehabilitation are not effective, the patient is qualified for surgical treatment, such as total hip arthroplasty (THA). Given its high reliability, this procedure was hailed as the “operation of the century” [2].

The success of the procedure depends both on the patient, in whom age, comorbidities and body mass index (BMI) are important, as well as on the surgeon responsible for implant orientation, proper surgical approach, and the selection of the type of implant [3,4,5].

Currently, the focus of arthroplasty development is on creating better implants with advanced designs, coatings, shapes, and materials used in implant alloys, alongside enhancing articulation and surgical techniques. The overarching aim of these efforts is to maximize the survival (biofunctionality) and functionality of the prosthesis within the patient’s body.

The L-Cup is made of a titanium alloy for cementless implantation and was manufactured by Biomet. It is a spherical bearing, available in the “spherical press-fit without fins”, “spherical press-fit with fins”, and “spherical threaded cup” versions. They were used from the late 1990s until around 2010. It is covered with titanium cladding produced using “plasma spray” technology. The outer diameter of the implant varies from 46 mm to 70 mm, with a pitch of 2 mm.

The L-Cup acetabular component, to the best of our knowledge, is the only threaded acetabular component for which the manufacturers recommend impaction during implantation. Our hypothesis is that combining the press-fit and screw-in methods during hip prosthesis implantation improves prosthesis survival outcomes by enhancing the stability of the acetabular component and reducing the risk of its migration in the long-term perspective.

The aim of the study was to analyze the outcomes of uncemented THA in patients with an L-Cup acetabular component implanted, with a follow-up period of at least 14 years and mean follow-up period of more than 19 years. The primary objective was to assess the survivorship of the acetabular component; the secondary objective was to assess clinical outcomes using the Merle d’Aubigné and Postel classification, modified by Charnley (MAP) and relief pain using the VAS score and radiology to find potential sign of osteolysis.

## 2. Materials and Methods

The permission for the study was granted by the Local Bioethics Commission (agreement no RNN/195/24/KE).

### 2.1. Patients

Between February 1999 and November 2010, 351 L-Cup acetabular components were implanted in 315 patients (36 bilateral cases) at our department (351/ 4472 cups implanted during this period).

**Figure 1:**
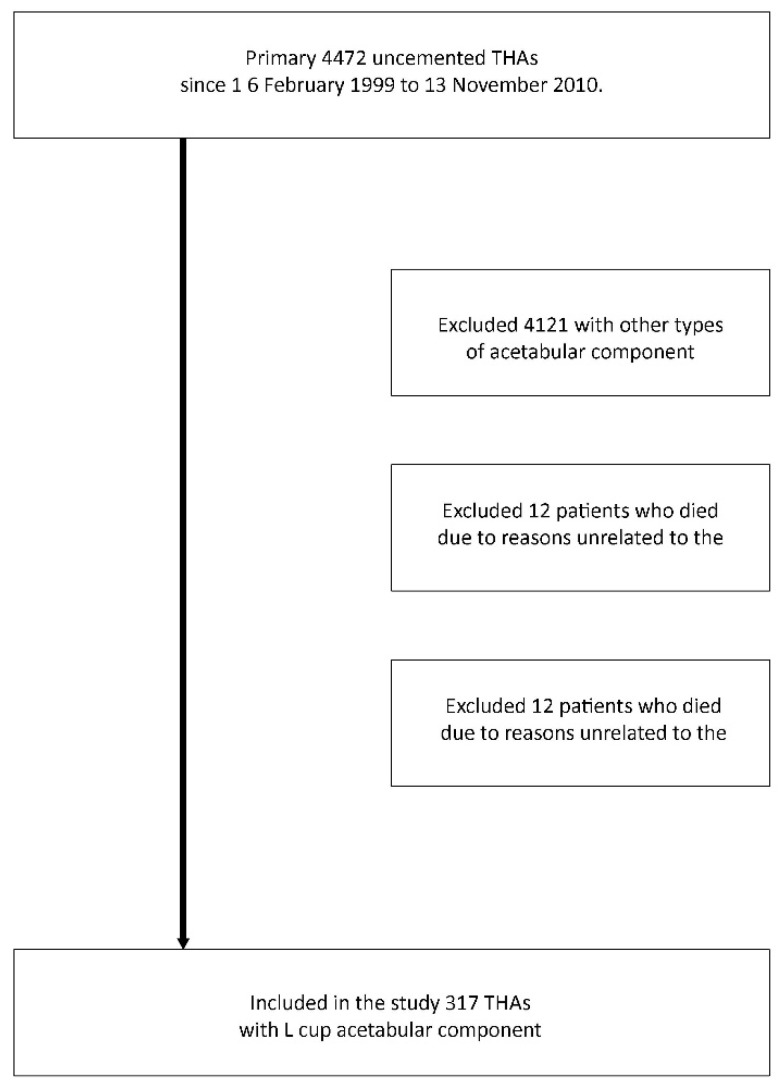
Flow diagram of the distribution of the patient population in the study. THA—total hip arthroplasty.

In our study group, surgical treatment was indicated for advanced idiopathic osteoarthritis of the hip joint, post-hip dysplasia (DDH), avascular necrosis of the hip (AVN), after trauma or post inflammatory. The inclusion criteria for the study were the presence of advanced pain symptoms and/or a limited range of motion in the hip joint that did not respond to conservative treatment. Preoperative X-rays were evaluated using the Kellgren–Lawrence classification system or Crowe classification in post-DDH cases [6,7]. The exclusion criteria included prior surgery on the acetabulum or proximal femur, morbid obesity (defined as BMI greater than 40), severe neurological or systemic conditions, or rheumatoid arthritis. During the follow-up, 12 deaths unrelated to the procedure were observed. In addition, 22 other patients did not report for the final examination and were therefore excluded from the study (Figure 1).

### 2.2. Design of the L-Cup Acetabular Component

**Figure 2:**
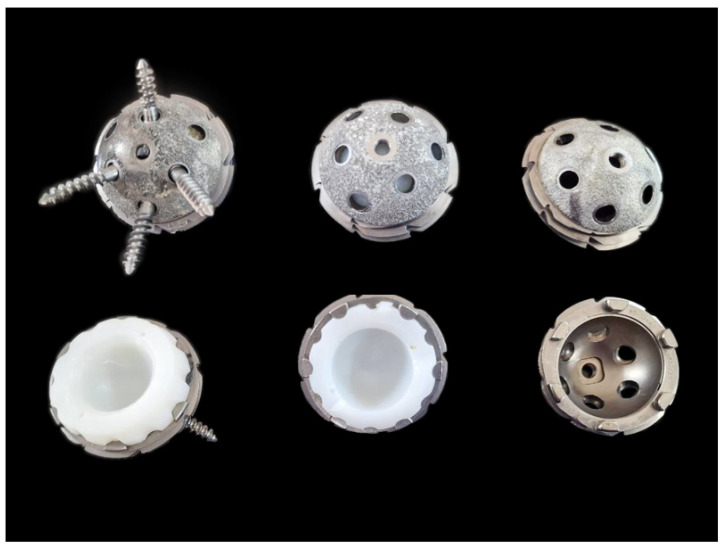
Design of L-Cup acetabular component.

The primary stability of L-Cup acetabular component is attained through its specific design and construction features. The subsequent integration of the cup into the bone ensures high secondary stability. The L-Cup acetabular component is made from a titanium alloy, which provides significant benefits due to their high elasticity and excellent biocompatibility. The porous titanium coating on the dome of the cup is applied using a plasma spray technique. This porous layer greatly enhances the surface area, creating optimal conditions for safe osteointegration and secondary stability. Each type of L-Cup acetabular component includes screw holes in the dome area, and the square connector with a central thread facilitates a secure attachment to the L-Cup acetabular component impactor (Figure 2).

The ringlock design ensures a consistent maximum strength of polyethylene for any combination of cup and inlay. The L-Cup acetabular component features a titanium ring that securely fits into the outer groove of the inlay, providing a strong anchorage. This connection between the inlay and the cup prevents potential micromovement and enhances rotational stability. A precise fit and continuous polyethylene strength help reduce contact stress and wear. The ringlock inlays are made from Arcom polyethylene.

An important aspect of the L-Cup acetabular component design were the holes intended for the possible implantation of stabilizing screws. These holes, if not used, did not have plugs.

### 2.3. Surgical Technique

All procedures were performed by experienced orthopedic specialists. Each procedure was performed in the prone position using an anterolateral approach. A second-generation cephalosporin was given 30 min before the operation. The acetabular component of the hip prosthesis was placed within the “safe zone” defined by Lewinnek [8]. In the majority of cases, the artificial acetabulum was positioned in the region corresponding to the anatomical location of the original acetabulum, referred to as the True Acetabular Region (TAR) [9].

### 2.4. Acetabular Implantation

Once the reaming process is finished, a threaded cup can be inserted. This cup is attached to the shaft of the insertion plate and carefully placed within the prepared acetabulum. Initially, the ratchet is connected to the shaft of the insertion plate, followed by attaching the corresponding handle. Prior to inserting the threaded cup, it is essential to stabilize the surrounding soft tissues to avoid injury from the threads. The instrument containing the cup is then inserted into the acetabulum at the appropriate angle, with the ratchet handle turned in a clockwise direction. To make any adjustments, it is turned counterclockwise. This procedure is repeated until the cup is fully inserted, ensuring it reaches the acetabular base. Once fully inserted, the edge of the cup should be aligned with the natural rim of the acetabulum.

If necessary, especially in cases of defects, the contact between the acetabulum and the implant can be enhanced by placing a cancellous graft in the prepared acetabulum before inserting the cup. If the cup is positioned too deeply horizontally or vertically, it needs to be repositioned, which can also be accomplished by using a 10 mm offset inlay. A slight resistance when pressing the inlay confirms its solid fit.

The L-Cup is, to our knowledge, the only threaded acetabular component for which the manufacturer’s recommended technique includes an initial striking by the surgeon.

### 2.5. Postoperative

Following surgery, all patients received a 30-day regimen of low-molecular-weight heparin for thromboprophylaxis in accordance with the guidelines of the Polish Society of Orthopedics and Traumatology [10]. One day post-surgery, they were permitted to bear weight as tolerated using a walker or crutches. The radiological follow-up consisted of an anteroposterior view of the pelvis and an axial view of the operated hip. The clinical condition and radiological evaluation were conducted 2 days after the procedure, after 3, and 12 months and then annually or sooner if the patient reported concerning symptoms.

The clinical outcomes were evaluated using the Merle d’Aubigné and Postel classification, as modified by Charnley (MAP) [11]. This method involves a point-based assessment of pain, gait, and the total range of passive motion in the operated hip joint. Pain was quantified using a ten-point Visual Analog Scale (VAS), where 0 represented no pain and 10 indicated the most severe pain experienced by the patient [12]. The positioning of the prosthesis was assessed, along with the degree of implant integration with the bone and the presence and extent of heterotopic ossification. Additionally, the horizontal, vertical, and angular migrations of the acetabular component were analyzed. The integration of the acetabular component was categorized using the De Lee and Charnley [13] three-stage classification. Notably, all radiological assessments were performed by an independent researcher who was not involved in the surgical procedures under analysis.

### 2.6. Statistical Analysis

Statistical analyses were conducted using IBM SPSS Statistics for MacOS, Version 29 (IBM Corp., Armonk, NY, USA). The nominal data from unpaired observations were compared using the Chi-square test, while McNemar’s test was utilized for paired observations. The Shapiro–Wilk test was employed to assess the normality of the data. A *p*-value of less than 0.05 was considered statistically significant.

## 3. Results

A total of 317 hips (182 females and 135 males), with a mean age of 60 ± 11.25 years at the time of total hip arthroplasty (THA), were included in the study. The mean follow-up period was 19.2 years (range 14–25 years). The patient demographics and preoperative diagnoses are presented in Table 1.

In the preoperative evaluation, the clinical findings were uniformly unfavorable across all cases. All hip joints were classified as grade IV according to the Kellgren–Lawrence scale [14].

In the study group, the following acute complications were observed. During the procedure, six periprosthetic fractures of the proximal femur occurred, which were treated with cerclage. For these patients, a protocol of slower postoperative rehabilitation was implemented to offload the affected limb. In three patients from the DDH group, dislocations occurred within 3 months of surgery. All dislocations were treated conservatively with immobilization in neutral rotation for 6 weeks. The outcome for each dislocation was positive. Additionally, seven cases of paresis were observed, affecting the femoral nerve in four instances and the fibular component of the sciatic nerve in three cases. During the follow-ups, these symptoms were completely resolved. No cases of thromboembolic complications or other deaths related to the surgery were observed in the study group.

During the procedures performed on patients under the age of 50, ceramic femoral heads were used, while metal heads were employed in older patients, in accordance with the standard practice at our clinic at the time. We did not compare the groups based on the type of femoral head, as these groups are heterogeneous. Nearly the entire post-DDH group received ceramic heads due to the earlier timing of their primary procedures. Therefore, we consider the classification and evaluation based on the type of femoral head to be non-objective.

In 120 out of 317 cases, we used the Taperloc Aura II stem and in 197 out of 317, we used the Exception stem. In total, 95.6% of the stems were seated correctly. In the remaining cases, a slight varus or valgus in the position was visible, which did not affect the patient’s clinical result or the time of revision.

The decision to use screws was made intraoperatively on a case-by-case basis. Screws were employed in 201 out of 317 cases. A single screw was never used; two screws were applied in 44 cases (both in the femoral shaft), while three screws were used in 157 cases (two in the femoral shaft and one in the ischium). The use of screws had no significant impact on the survival of the acetabular component. There was no correlation between the loosening rate and the presence or number of screws used.

Patient VAS scores were collected before and after THA. The mean preoperative score was 7.2 points, which improved to 2.1 points after hip replacement, indicating a statistically significant improvement. The most significant benefits were observed in reduced or eliminated pain and an increased range of motion in the operated joint, leading to a greater overall satisfaction with the procedure [12].

**Figure 3:**
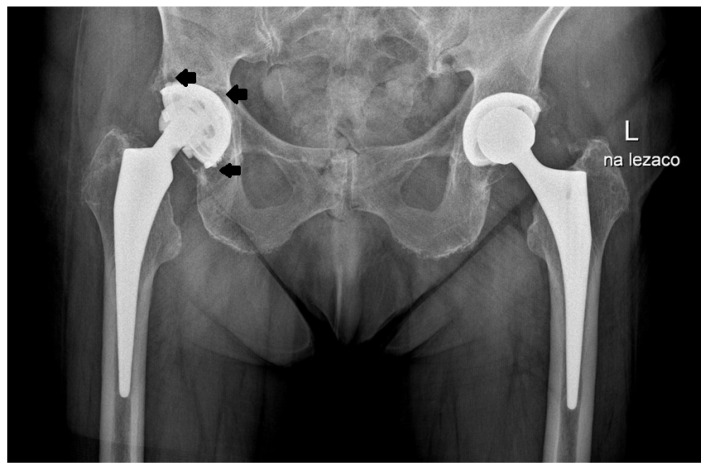
X-ray images of a patient who qualified for right hip revision surgery. The arrows indicate the loosening of the acetabular element.

**Figure 4:**
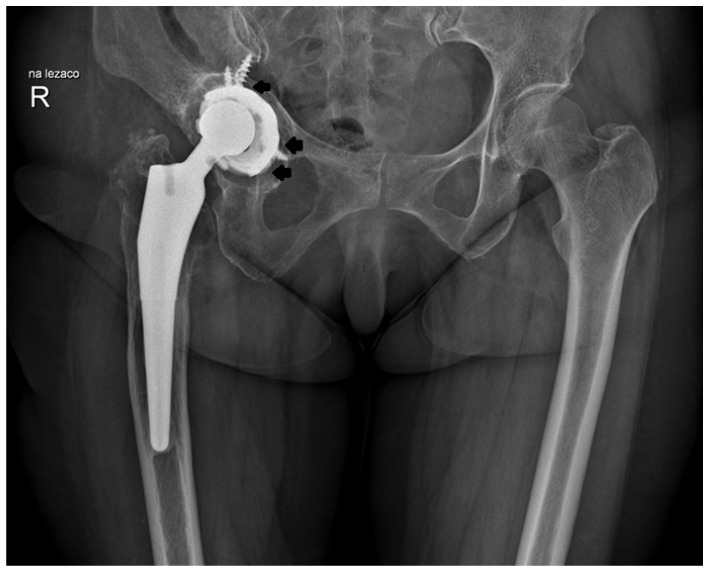
X-ray images of patient who qualified for right hip revision surgery. The arrows indicate the loosening of the acetabular element.

**Figure 5:**
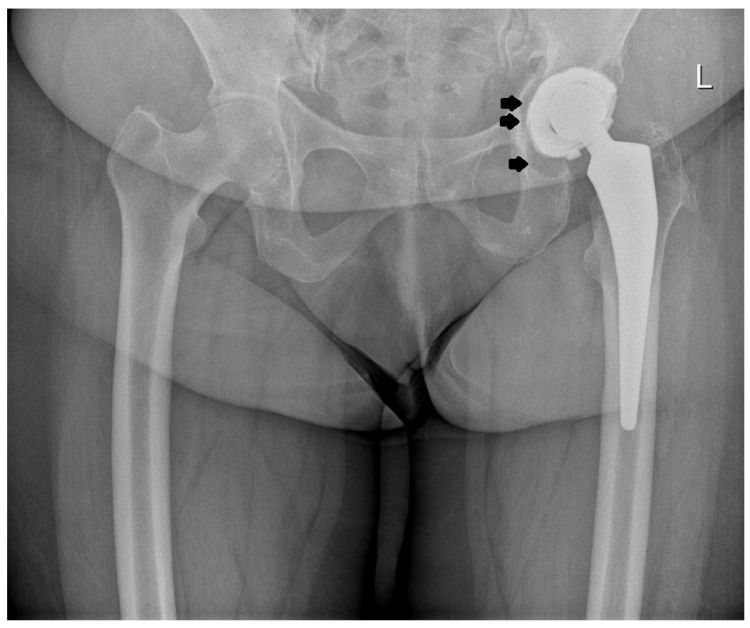
X-ray images of patient who qualified for left hip revision surgery. The arrows indicate the loosening of the acetabular element.

**Figure 6:**
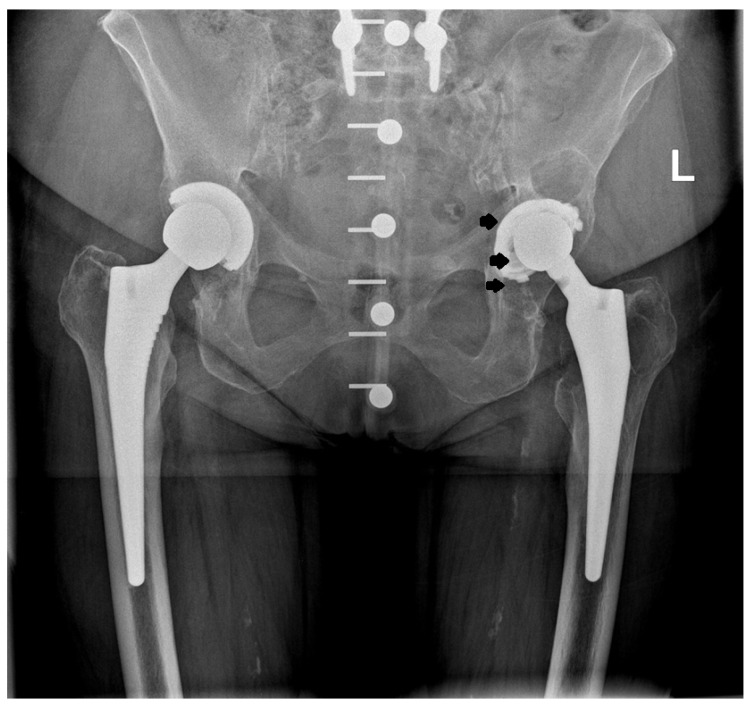
X-ray images of patient who qualified for left hip revision surgery. The arrows indicate the loosening of the acetabular element.

Prior to the procedure, all patients were evaluated, and their results were consistently poor; however, their subjective assessments following surgery were markedly more favorable compared to the outcomes based on the modified MAP classification [11]. The notable improvements included a marked reduction or complete resolution of pain and an increased range of motion in the operated joint, which contributed to high overall satisfaction with the procedure. As anticipated, the outcomes were somewhat less favorable in patients treated for coxarthrosis secondary to developmental dysplasia of the hip (DDH). However, it is important to note that a rating of “excellent” on the modified MAP scale reflects a result comparable to that of a healthy hip joint. Over an average follow-up period of 19 years after surgery, the improvement in the final clinical assessment, based on the modified MAP classification, was 6.3 points, indicating a statistically significant improvement. The results showed the following: excellent in 157 cases (49.5%), good in 64 (20.2%) cases, satisfactory in 31 cases (9.7%), and poor in 65 cases (20.5%). All poor outcomes were related to the loosening of the prosthesis. The MAP results based on the different etiologies of the procedure are presented in Table 2 while the examples of the X-rays of patients who qualified for revision surgery are shown in Figure 3, Figure 4, Figure 5 and Figure 6.

Among the 317 patients, 65 revisions were performed. Of these, 61 were due to the isolated loosening of the component, while 4 involved the loosening of both the acetabular component and the femoral stem. Additionally, two of the revisions were caused by septic complications that occurred more than one year after the primary surgery. In both cases, the treatment began with targeted antibiotic therapy, followed by a two-stage surgical procedure involving the use of a spacer, which was successfully completed. Each revision case was classified as poor according to the modified MAP classification.

The Kaplan–Meier estimator was used to calculate the probability of implant survival based on the results obtained [15]. The 5-year biofunctional survival rate was evaluated for 317 cases: 97.15% for the entire prosthesis, 97.78% for the acetabular component and 99.37% for the femoral stem. The 10-year biofunctional survival rate was assessed for 317 cases, yielding 88.92% for the total prosthesis, 90.51% for the acetabular component, and 98.42% for the femoral stem. The 15-year Kaplan–Meier rates for 266 cases were 77.07% for the total prosthesis, 80.45% for the acetabular component, and 96.62% for the femoral stem. The 20-year rate was evaluated for 103 cases: 71.84% for the entire prosthesis, 73.78% for the acetabular component and 98.06% for the femoral stem. A detailed summary of the survival results for the prosthesis according to Kaplan–Meier is presented in Table 3 [15].

The study group was divided into three subgroups based on BMI, which was assessed on the day of hospital admission prior to the primary THA procedure. The first group, with a BMI of up to 29.9, included 147 out of 317 patients; the second group, with a BMI between 30 and 34.9, included 154 out of 317 patients; and the third group, with a BMI between 35 and 39.9, included 16 out of 317 patients. We did not observe any statistically significant differences in prosthesis survival between these groups.

## 4. Discussion

The aseptic loosening of the acetabular component over time remains a significant challenge after THA. The careful selection of the appropriate implant during preoperative planning is crucial [16].

Historically, two key factors have been recognized for the long-term success of uncemented implants: (1) achieving primary stability through a secure bone–implant connection with limited micro motion, and (2) ensuring secondary osteointegration for lasting stability [17,18].

The first generation of threaded cups was designed with smooth surfaces, based on the belief that direct mechanical contact between bone and the metal shell would ensure sufficient primary and long-term fixation [19,20,21,22,23].

In a clinical and radiographic study by Fernandez-Gonzales et al. [22], 60 cementless threaded Lord cups were evaluated over an average follow-up period of 6 years. The results showed that 18% of the cups required revision, while 43% demonstrated fibrous stable fixation. Similarly, Clarius et al. [24] reported a 49% survival rate for the cups after 17 years. Fink et al. [23] found that 73% of Link type V threaded cups experienced a migration greater than 3 mm and tilting over 5°, with a cumulative survival rate of 70.2% after 15 years [25]. The poor performance of this generation of cups has been extensively documented in the literature [20,25,26,27].

It quickly became apparent that primary fixation alone was insufficient to ensure the long-term durability of the implant, as elevated rates of loosening were observed in the medium term. Therefore, the establishment of biological fixation was deemed necessary to achieve favorable long-term outcomes. For this reason, second- and third-generation threaded cups were introduced [28]. Second-generation cups were characterized by a porous or a hydroxyapatite (HA) coating on the metal surface while in the third-generation cups, modular inlays were used. The implementation of these modifications led to enhanced implant survival rates [28].

Epinette et al. [29] reported a survivorship of 99.43% at 10 years based on 276 hips. The cup used in this study was the Arc2f cup coated with hydroxyapatite using the plasma spray process [29]. Subsequently, Schuh et al. [30], in their study of the Wagner conical screw, obtained a 93.2% survival rate at the 11.5-year follow-up. Zweymuller et al. [31], in their study based on a threaded double-cone cup without additional screw fixation, observed a 98.6% survival rate at the 10-year follow-up. Pellengahr et al. [32] observed similar results in a study based on the Munich II threaded cup (second generation). This study described the postoperative results of 53 patients with a mean follow-up period of 7 years and 11 months. Moreover, they also observed a notable enhancement in both clinical and radiographic outcomes when compared to the Munich type I threaded ring (first generation) [32]. The last two described cups also had an additional advantage over their predecessors due to the lack of screw holes. They were not used in these models without consequences in the above degree of aseptic loosening. The lack of screw holes solved the problem occurring in the earlier threaded cups, which was osteolysis [33].

Periprosthetic osteolysis is a key problem in THA [33]. It refers to excessive bone loss primarily caused by polyethylene particles that provoke an unfavorable reaction in the surrounding bone [34]. Essentially, the numerous particles generated from the joint surfaces enhance the maturation and lifespan of osteoclasts, promote the release of metalloproteinases, and lead to the formation of joint fluid, all contributing to the degradation of the bone around the implant. Consequently, wear contributes to particle disease, and a direct correlation has been suggested between the rate of wear and the extent of bone loss [35]. According to the study by Dumbleton et al., a critical wear rate is identified as exceeding 0.1 mm annually. This threshold correlates to volumetric wear rates of 62 mm³ and 80 mm³ per year for 28 mm and 32 mm femoral heads, respectively. These values are regarded as the “osteolysis threshold” [36]. The occurrence of osteolysis was first described in a study of ABG I cups by Galloa et al. [37] The average wear for this socket was 0.363 mm per year. In their study, Nieuwenhuis et al. [38] investigated the effect of drill holes in threaded cups, focusing on the Omnifit cup with an average follow-up of 60 months. The results revealed a substantial 43% incidence of acetabular osteolysis. The authors suggested a direct relationship between the degree of backside wear and the number of screw holes present in the threaded cup [38]. In the group we studied, osteolytic changes in the area of the openings were observed in 78% of the loosened cups. Additionally, in 43 patients in whom there was no loosening of the cup, we observed minor changes on their X-ray images. These patients showed no clinical symptoms, were informed of this fact, and a decision for a watchful waiting approach was made in their case. We must remember that a significant drawback of the L-Cup acetabular component is the lack of plugs for the screw holes, where osteolytic changes are most pronounced. Nevertheless, there is no definitive evidence regarding the efficacy of screw hole plugs [39].

An undoubted advantage of the study we conducted is the large group of patients and long follow-up. Tindal et al. [40] described excellent results of 112 threaded cups. The study found that the 13-year survival rate of the JRI HAC-coated threaded acetabular cup is 99% [40]. Another study by Clarius et al. [16] reported Weill cup results based on a group of 127 hips. According to that study, the 17-year Kaplan–Meier indicator for acetabular revision due to aseptic loosening was 78% [16]. Datir et al. [41] reported that the survival rate of the HAC-coated threaded cups was 94.1 at 15 years in a study based on 108 hips. They also reported a polyethylene consumption rate of (0.24 mm/year) without evidence of osteolysis [41]. Almeida et al. [42], in their study based on 202 hips with an average follow-up of 16.9 years, described the threaded Tropic acetabular cup. They reported a Kaplan–Meier survivorship rate of 86.3% for the mechanical or radiographic loosening of the cup. In addition, they observed massive polyethylene wear in 56% of patients. For the above reasons, the authors discarded the idea of a threaded cup design and opted for a press-fit acetabular cup instead [42].

### Limitations

Several limitations should be acknowledged in this study. First, the data collection spanned a long period, from 1999 to 2010, during which there were advances in both surgical techniques and implant technology. The uncemented L-Cup component, used in this study, was introduced in a period when the understanding of optimal implant materials and fixation methods was still evolving. Over time, there has been a shift toward more advanced coatings, better polyethylene materials, and modifications in implant designs to reduce wear and loosening rates. This could affect the relevance of the results for current practice, as the L-Cup’s performance may not reflect the outcomes of more recent implant designs. Second, the experience of the surgical center, which gained considerable expertise over the study period, could introduce variability in the results. Changes in the surgical team’s experience and the development of improved surgical techniques during the follow-up period may have influenced the clinical outcomes. Additionally, the lack of a uniform approach to post-surgical care and rehabilitation protocols might have contributed to the variability in the recovery process. Another limitation is the relatively long follow-up period (ranging from 14 to 25 years), which, while a strength in terms of assessing long-term outcomes, also means that patient data were affected by the passage of time. Several patients did not attend the final assessments or were lost to follow-up, potentially introducing a bias toward the more successful outcomes in the surviving cohort. Furthermore, the inclusion of a broad age range and different etiologies of hip arthritis (idiopathic OA, post-DDH, AVN, etc.) may contribute to heterogeneous results, complicating the interpretation of the outcomes across all subgroups. This study was conducted on a specific patient population from a single center and may not be representative of other populations, particularly those with different demographic characteristics, comorbidities, or access to advanced healthcare. Therefore, the generalizability of the results to other settings or broader populations remains limited. Finally, while the study provides valuable data on implant survival and clinical outcomes, it is a retrospective analysis, meaning that it is subject to the limitations inherent in this type of study design, including the possibility of selection bias and reliance on historical data. Further prospective studies with larger, more diverse cohorts and modern implant technologies are needed to confirm these findings and provide more robust conclusions.

## 5. Conclusions

THA is an effective treatment for advanced degenerative joint diseases of various etiologists. One of the key factors, alongside proper patient qualification, preoperative planning, and surgical technique, is also the selection of implants. In our study, the L-Cup acetabular component loosened in 20.5% of cases, suggesting that the technique proposed by the producer, which involves both impacting and screwing the implant, was not successful in the long-term follow-up.

## Figures and Tables

**Table 1:** Patient demographics and preoperative diagnoses.

Parameter	Value
Demography	
Mean age	60 years (range 24–86)
Male/female	135/182
Right/left	162/155
BMI	32.4 (range 21.5–38.6)
Follow-up	19.2 years (range 14–26)
Preoperative diagnoses	
Idiopathic	181 (57%)
Post-hip dysplasia (DDH)	68 (21.5%)
Avascular necrosis of the hip	33 (10.5%)
Post-trauma	27 (8.5%)
Post-inflammation	8 (2.5%)

**Table 2:** Final outcomes according to Merle d’Aubigné and Postel, modified by Charnley classification compared between different etiologies. * *p*-value < 0.05 are considered significant.

MAP Score	Idiopathic	DDH Group	AVN Group	Post-Traumatic Group	Inflammation Group	*p*-Value
excellent	105 (33.1%)	6 (1.9%)	21 (6.6%)	14 (4.4%)	1 (0.3%)	<0.001 *
good	13 (4.1%)	29 (9.1%)	11 (3.4%)	8 (2.5%)	3 (0.9%)	0.434
satisfactory	19 (5.9%)	6 (1.8%)	1 (0.3%)	4 (1.2%)	1 (0.3%)	0.445
poor	34 (10.7%)	27 (8.5%)	0	1 (0.3%)	3 (0.9%)	<0.001 *

**Table 3:** The Kaplan–Meier biofunctionality coefficient for implants after 5, 10, 15, and 20 years of observation (95% confidence interval).

	Kaplan–Meier 5-Year Follow–Up (316 Hips)	Kaplan–Meier 10-Year Follow–Up (316 Hips)	Kaplan–Meier 15-Year Follow–Up (266 Hips)	Kaplan–Meier 20-Year Follow–Up (103 Hips)
Both elements	97.15% (99.012–95.29)	88.924% (92.593–85.54)	77.067% (82.822–71.312)	71.84% (82.092–61.597)
Cup	97.78% (99.425–96.143)	90.50% (93.903–87.109)	80.45% (85.764–75.137)	73.79% (83.674–63.898)
Femoral stem	99.37% (100.244–98.489)	98.42% (99.804–97.030)	96.62% (98.827–94.406)	98.06% (100.74–95.367)

## Data Availability

The original contributions presented in this study are included in the article. Further inquiries can be directed to the corresponding author.

## Abbreviations

THA—total hip arthroplasty; OA—osteoarthritis; DDH—developmental dysplasia of the hip; AVN—avascular necrosis; SD—standard deviation, BMI—body mass index, VAS—Visual Analog Scale, MAP—Merle d’Aubigné and Postel, modified by Charnley.
